# Supplementary figures and images for: Methods for mediation analysis with high-dimensional DNA methylation data: Possible choices and comparisons
Source: PLoS Genet. 2023 Nov 7;19(11):e1011022. doi: 10.1371/journal.pgen.1011022 (PMC10655967; doi:10.1371/journal.pgen.1011022)

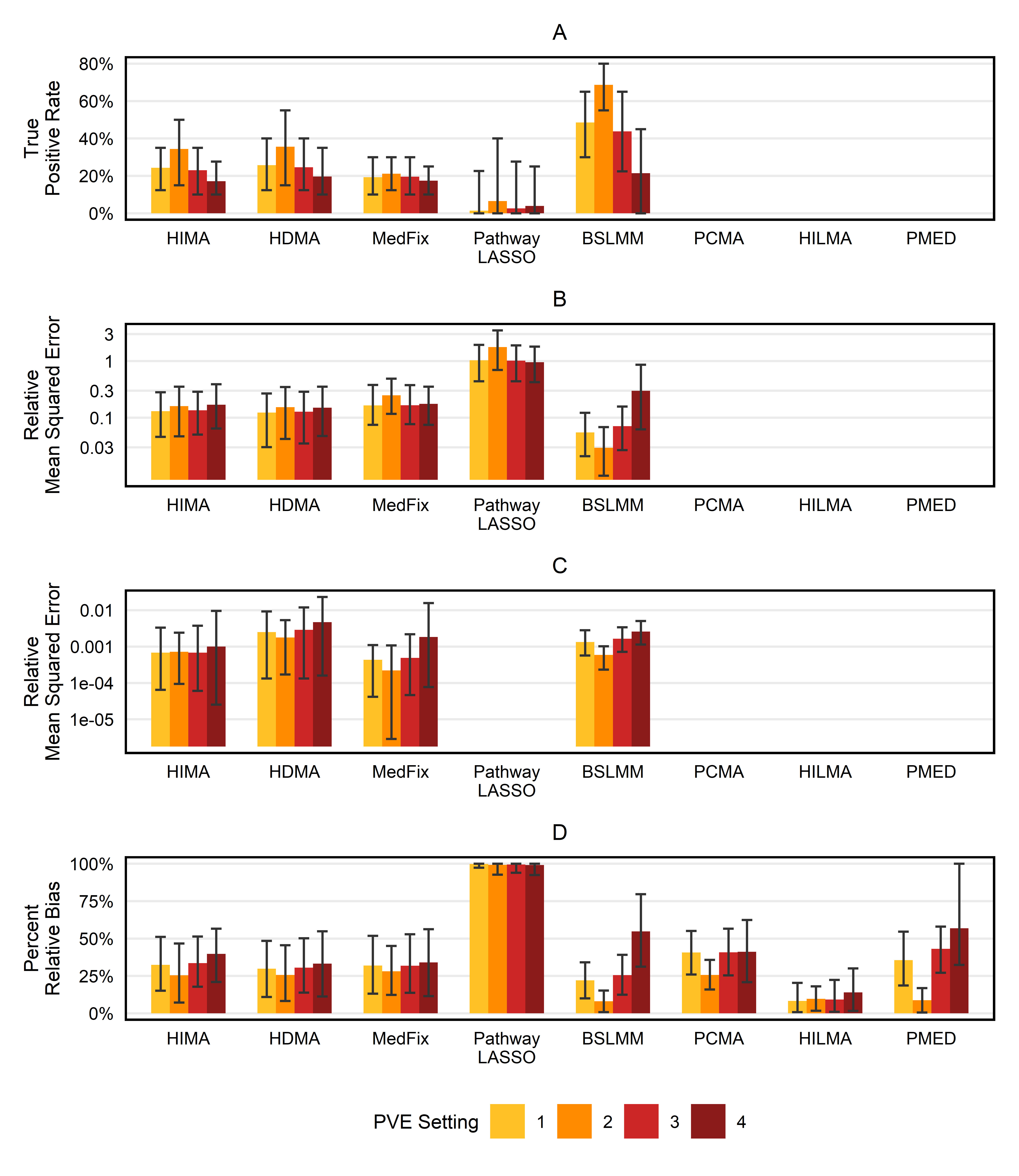

Supplement: S1 Fig — (A) True positive rate for detecting active mediators. (B) Relative mean squared error for estimating the mediation contributions of active mediators, relative to the one-at-a-time method. (C) Relative mean squared error for estimating the mediation contributions of inactive mediators, relative to the one-at-a-time method. (D) Percent relative bias for inferring the global mediation effect. The simulation settings for were created by taking the absolute values of the exposure-mediator and mediator-outcome effects in the original baseline simulation settings, which had four different proportion-of-variance-explained (PVE) settings: (1) PVEA = 0.2, PVEDE = 0.1, PVEIE = 0.1; (2) PVEA = 0.1, PVEDE = 0.1, PVEIE = 0.1; (2) PVEA = 0.2, PVEDE = 0.05, PVEIE = 0.1. (4) PVEA = 0.2, PVEDE = 0.1, PVEIE = 0.05. (PNG) [file pgen.1011022.s005.png]

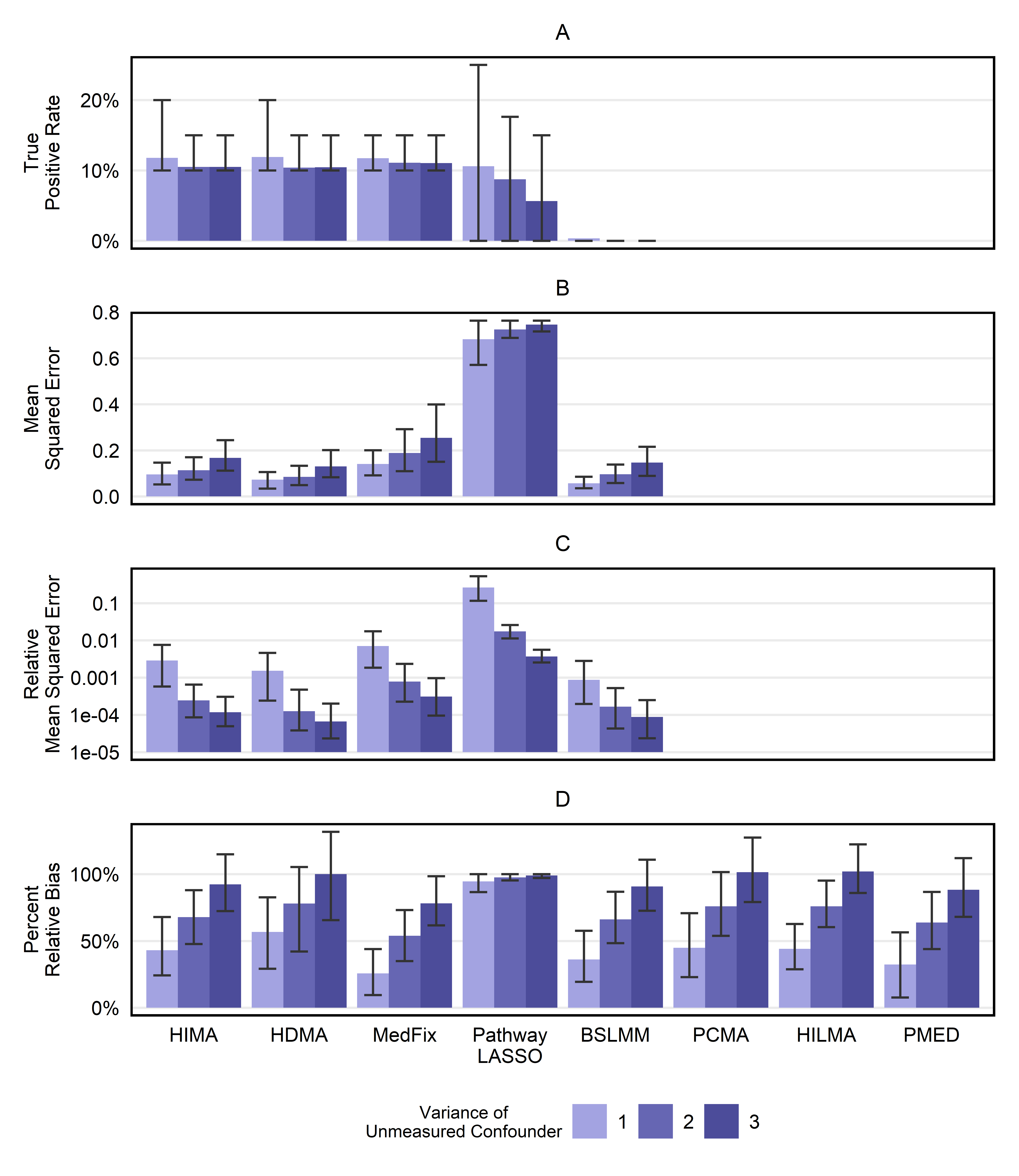

Supplement: S2 Fig — (A) True positive rate for detecting active mediators. (B) Mean squared error for inferring the mediation contributions of active mediators. (C) Relative mean squared error for inferring the mediation contributions of active mediators, relative to the one-at-a-time method. (D) Percent relative bias for inferring the global mediation effect. (PNG) [file pgen.1011022.s006.png]

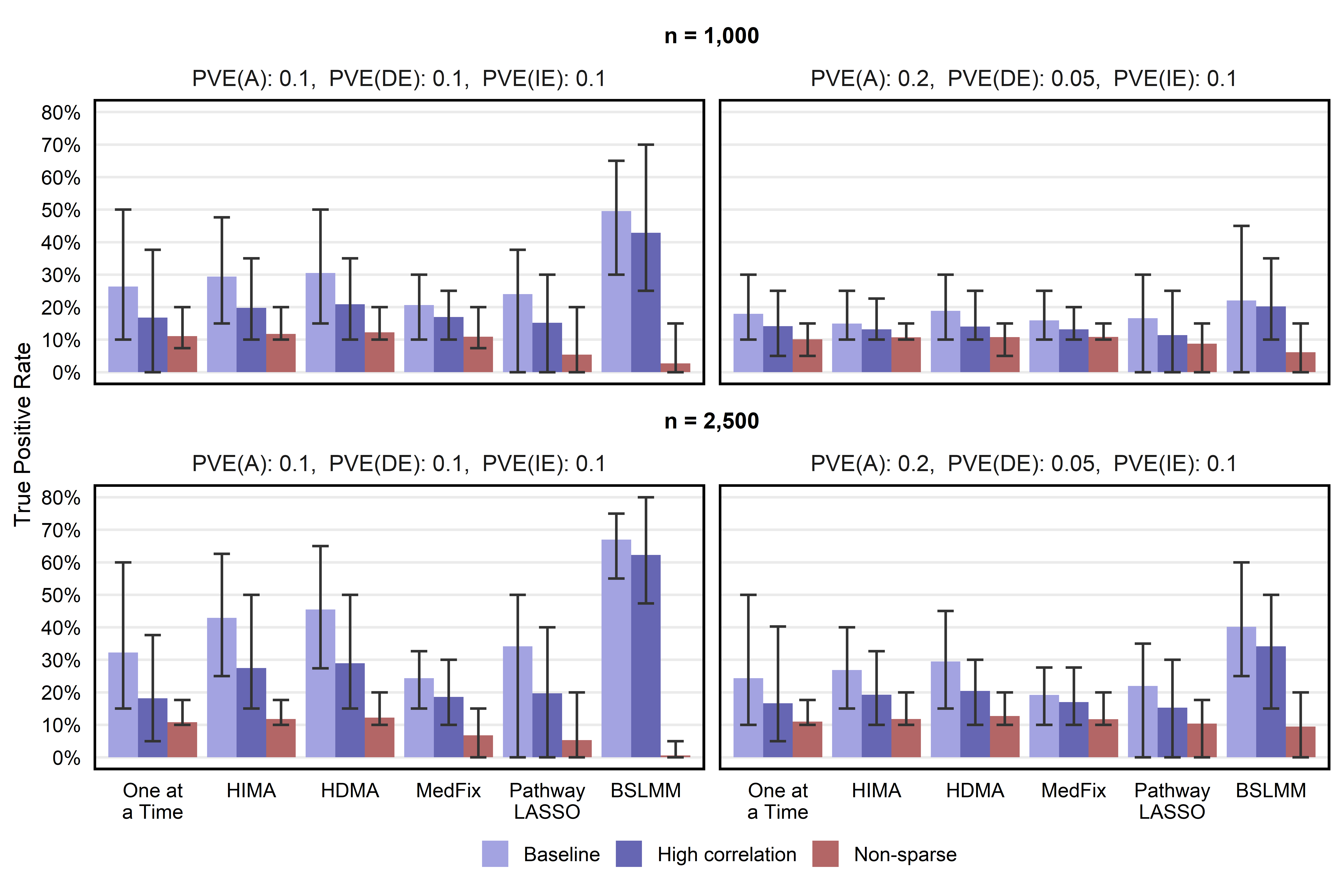

Supplement: S3 Fig — Mean true positive (TPR) rate and 95% empirical confidence interval for detecting active mediators in 100 simulated datasets. In the baseline and high-correlation-among-mediators settings, TPR is for distinguishing mediators which contribute to the global mediation effect from those which do not, whereas in the non-sparse setting, where all mediators contribute, TPR is for distinguishing mediators whose contributions were sampled from a high-variance distribution from those whose contributions were sampled from a low-variance distribution. False discovery rate was capped below 10% by a proper choice of the p-value threshold (one-at-a-time, HIMA, HDMA, MedFix), posterior inclusion probability threshold (BSLMM), or method tuning parameter (P-LASSO). PVE(A): Percent of variance in Y explained by the exposure. PVE(IE): Percent of variance in Y explained by the indirect effect. PVE(DE): Percent of variance in Y explained by the direct effect. (PNG) [file pgen.1011022.s007.png]

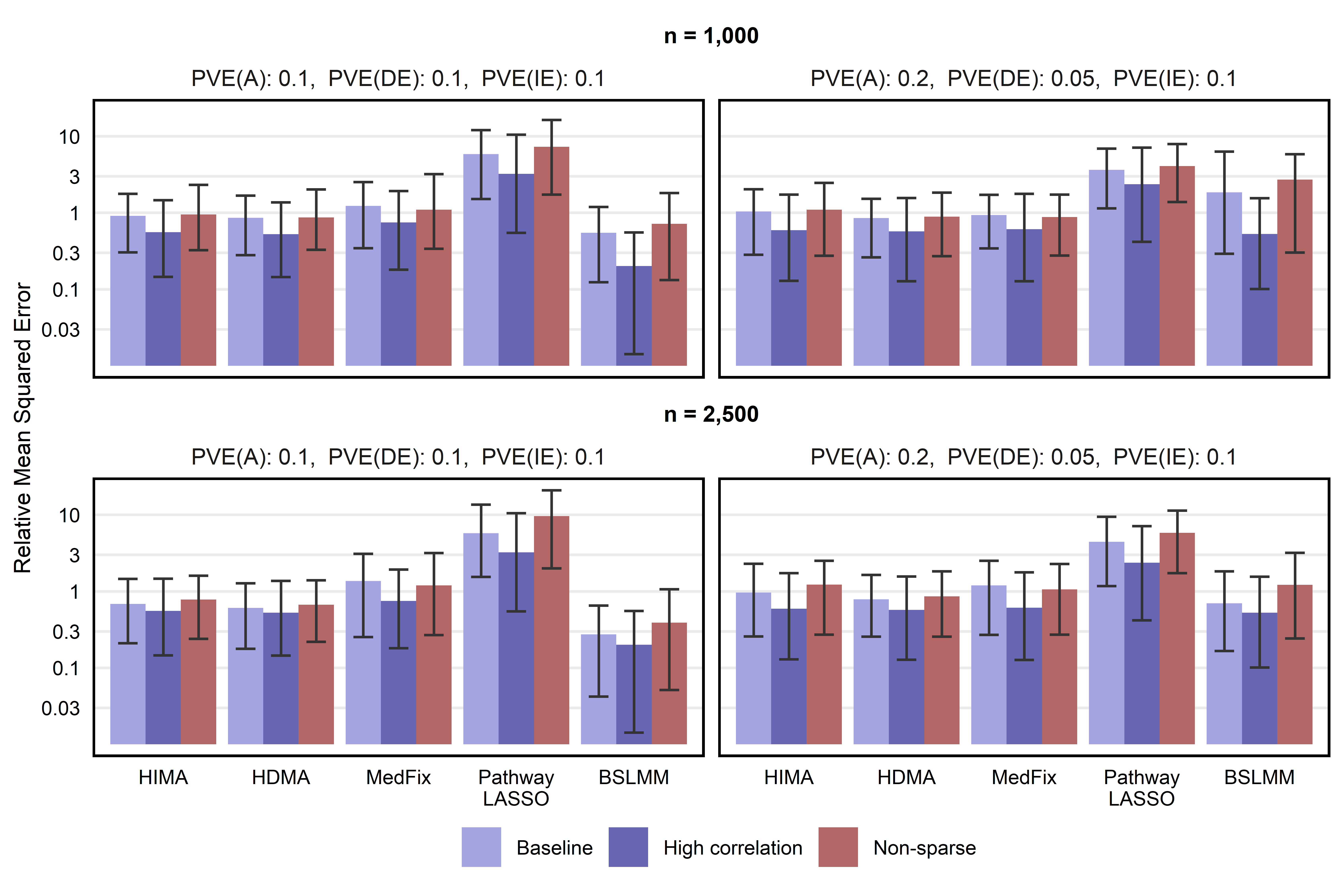

Supplement: S4 Fig — Mean relative mean squared error (rMSE) and 95% empirical confidence interval for estimating mediation contributions among active mediators in 100 simulated datasets, relative to the one mediator at a time method. Y-axis is on a log10 scale. For the baseline and high-correlation-between-mediators settings, active mediators are those which contribute to the global mediation effect, whereas in the non-sparse setting, where all mediators have some contribution, active mediators are those whose contributions were sampled from a distribution with large variance instead of small. PVE(A): Percent of variance in Y explained by the exposure. PVE(IE): Percent of variance in Y explained by the indirect effect. PVE(DE): Percent of variance in Y explained by the direct effect. (PNG) [file pgen.1011022.s008.png]

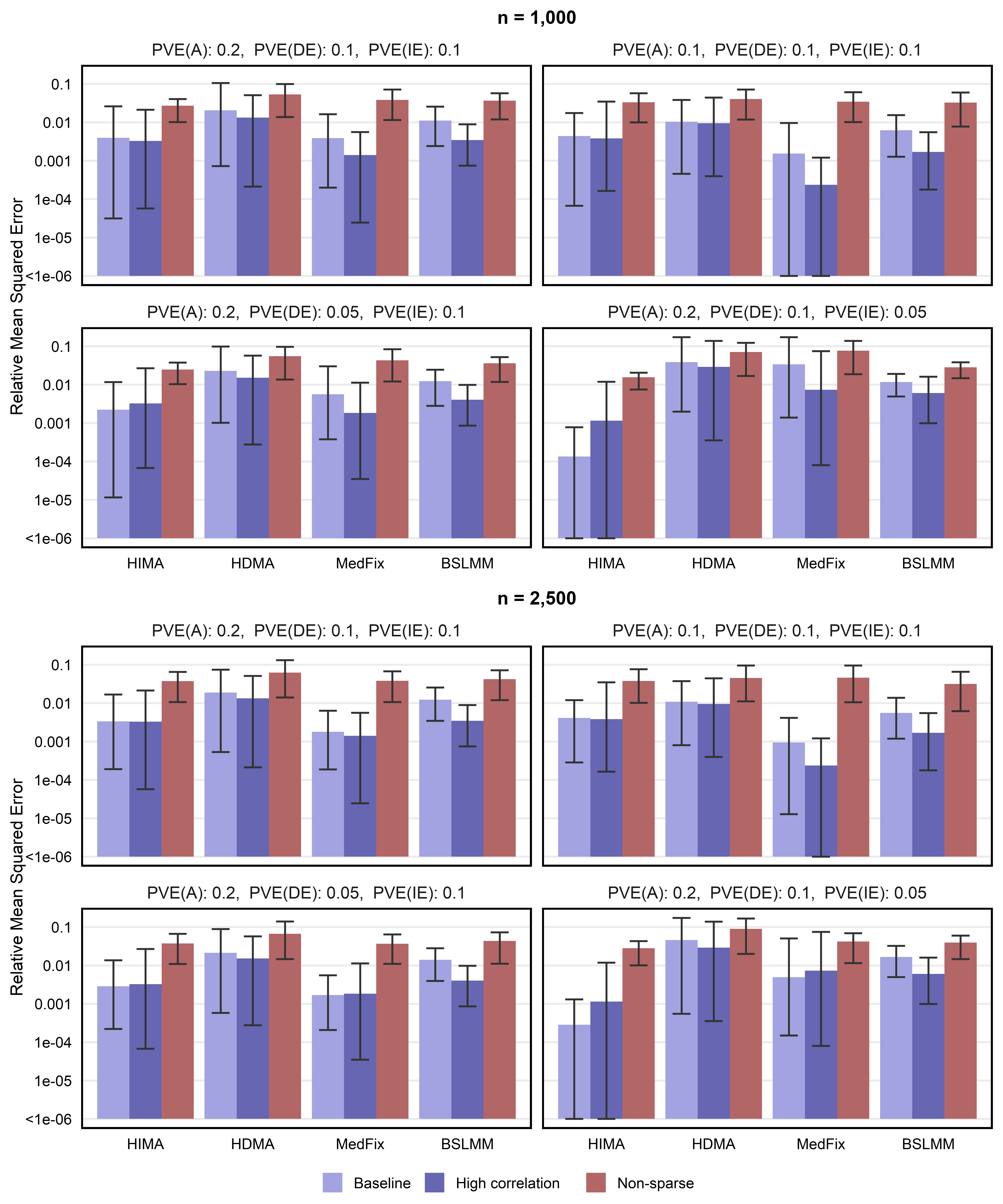

Supplement: S5 Fig — Mean relative mean squared error (rMSE) and 95% empirical confidence interval for estimating mediation contributions among inactive mediators in 100 simulated datasets, relative to the one mediator at a time method. Y-axis is on a log10 scale. For the baseline and high-correlation-between-mediators settings, inactive mediators are those which have no mediation contribution, whereas in the non-sparse setting, where all mediators have some contribution, inactive mediators are those whose contributions were sampled from a distribution with small variance instead of large. The method pathway LASSO is excluded from this figure because for multiple settings it had rMSEs of exactly zero. This happened because pathway LASSO tended to be highly conservative and successfully assigned inactive mediators to have no effect. PVE(A): Percent of variance in Y explained by the exposure. PVE(IE): Percent of variance in Y explained by the indirect effect. PVE(DE): Percent of variance in Y explained by the direct effect. (PNG) [file pgen.1011022.s009.png]

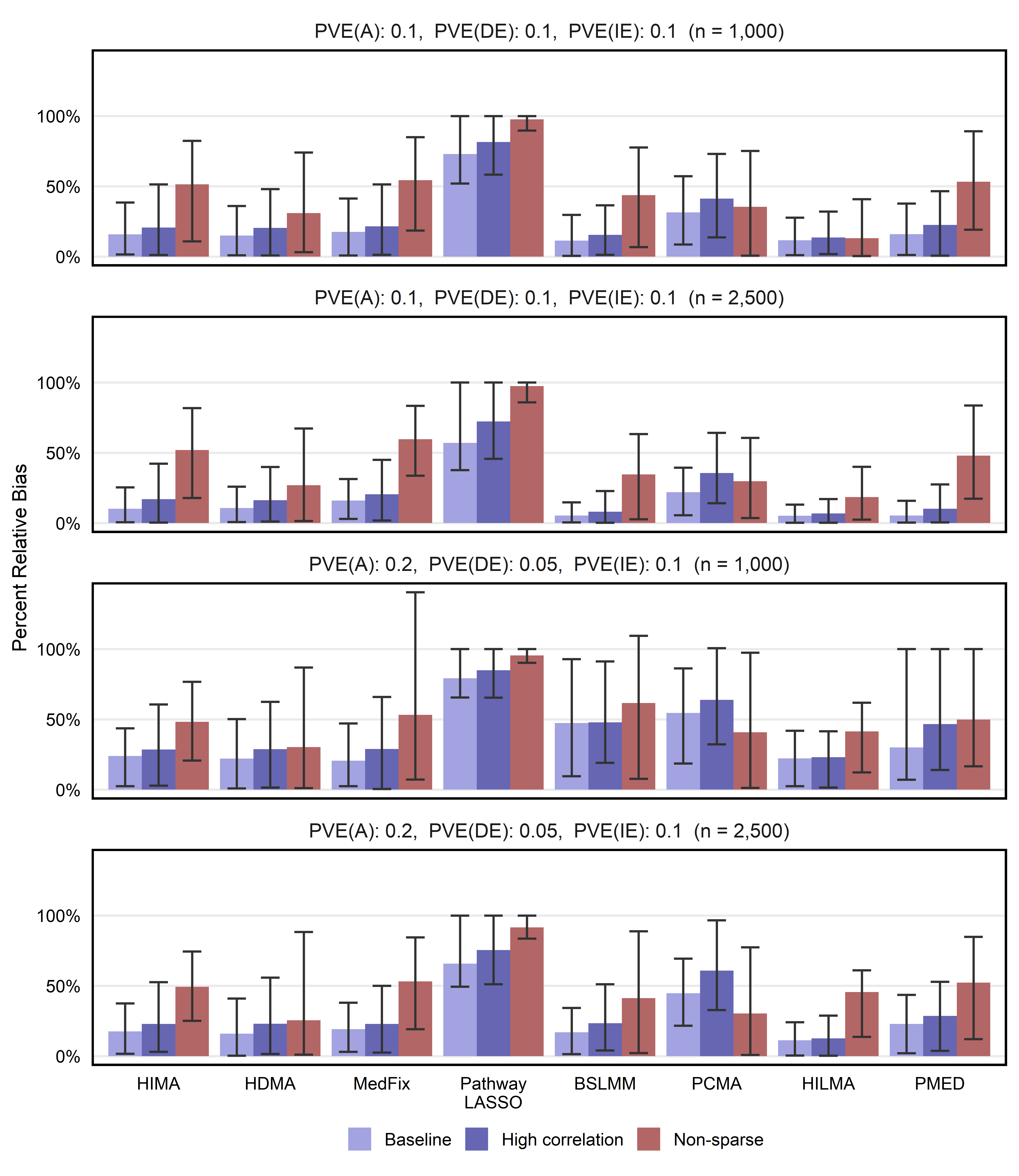

Supplement: S6 Fig — Mean percentage relative bias in estimating the global mediation effect across 100 simulated data replicates, with intervals representing the inner 95% range. PVE(A): Percent of variance in Y explained by the exposure. PVE(IE): Percent of variance in Y explained by the indirect effect. PVE(DE): Percent of variance in Y explained by the direct effect. (PNG) [file pgen.1011022.s010.png]

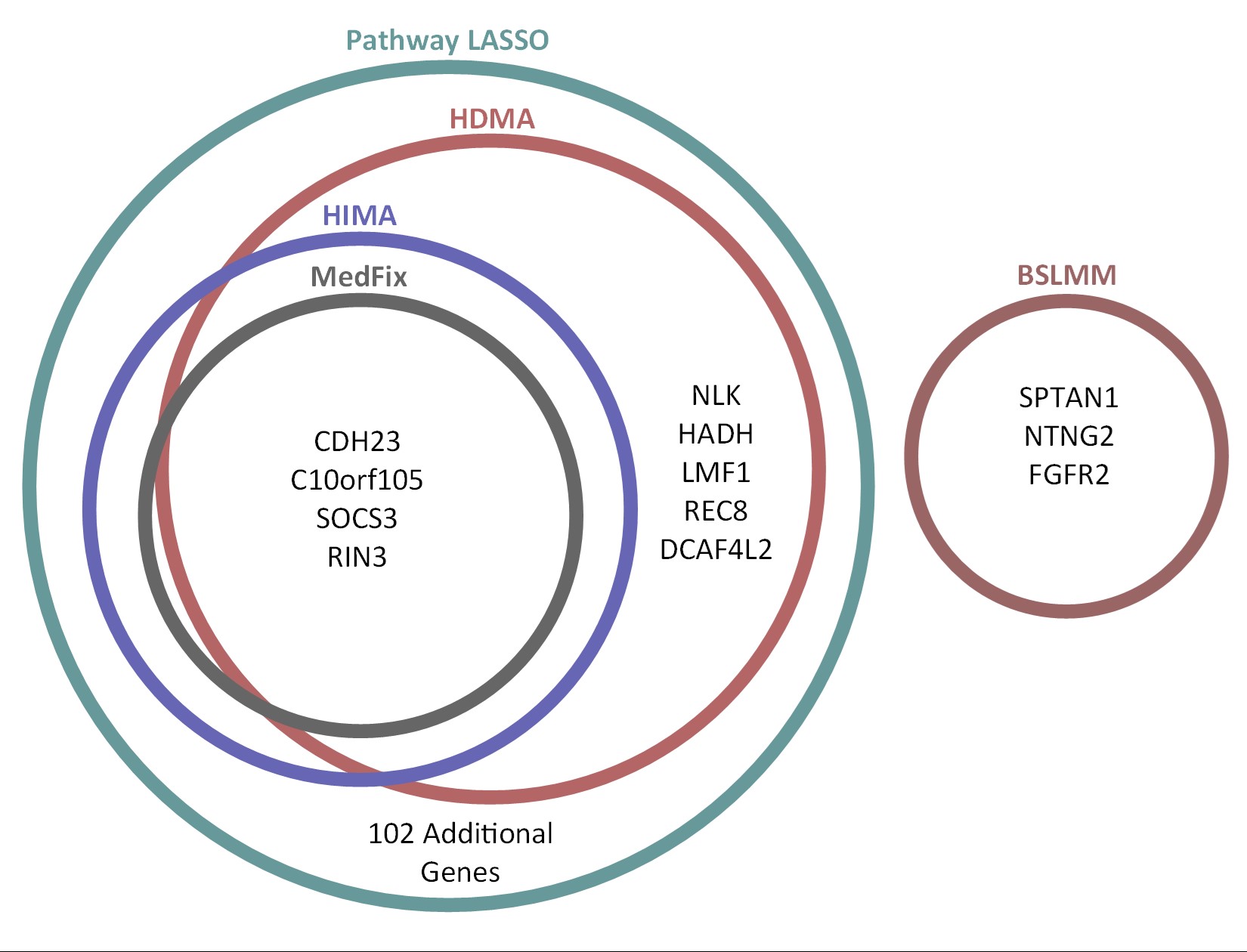

Supplement: S7 Fig — CpG sites were linked to genes using R Bioconductor package “IlluminaHumanMethylation450kanno.ilmn12.hg19”. Additional genes detected by Pathway LASSO listed in supplementary S1 File. (JPG) [file pgen.1011022.s011.jpg]
