# Supplementary material for: Methods for mediation analysis with high-dimensional DNA methylation data: Possible choices and comparisons
Source: PLoS Genet. 2023 Nov 7;19(11):e1011022. doi: 10.1371/journal.pgen.1011022 (PMC10655967; doi:10.1371/journal.pgen.1011022)
Supplement: S1 Table — (PDF) [file pgen.1011022.s012.pdf]

S1 Table. Summary of methods for high-dimensional mediation analysis.

Name and Citation	Estimation of global indirect effect	Estimation of mediation contributions	Active mediator identification	Y Data Type	Directly Allows Covariates	Summary
Group 1 Methods						
HIMA (1)	Point estimation	Point estimation	Yes	Continuous or binary	Yes	Fits the outcome model with the minimax concave penalty. Requires subsequent fitting of ordinary least squares regression to test the statistical significance the mediation contributions.
HDMA (2)	Point estimation	Point, interval estimation	Yes	Continuous or binary	Yes	Fits the outcome model with the de-sparsified LASSO penalty.
MedFix (3)	Point estimation	Point, interval estimation	Yes	Continuous	Yes	Fits the outcome model with the adaptive LASSO penalty. Can also be applied when the exposure is high-dimensional in addition to the mediators.
Pathway LASSO (4)	Point estimation	Point estimation	Yes	Continuous	No	Fits the outcome model and mediator models with a jointly penalized likelihood, directly applying shrinkage to the mediation contributions $(\boldsymbol{\alpha}_a)_j(\boldsymbol{\beta}_m)_j$.
BSLMM (5)	Bayesian point, interval estimation	Bayesian interval estimation	Yes	Continuous	Yes	Bayesian mixed-model in which the mediator-outcome associations $(\boldsymbol{\beta}_m)_j$ and the exposure-mediator associations $(\boldsymbol{\alpha}_a)_j$ are assumed to independently follow sparse normal distributions.
GMM (6)	Bayesian point, interval estimation	Bayesian interval estimation	Yes	Continuous	Yes	Bayesian mixed-model in which the mediator-outcome associations $(\boldsymbol{\beta}_m)_j$ and the exposure-mediator associations $(\boldsymbol{\alpha}_a)_j$ are assumed to jointly follow a sparse multivariate normal distribution.
Group 3 Methods						
PCMA (7)	Point, interval estimation	No	No	Continuous or binary	No	Applies principal component analysis on the mediator model residuals, transforming the mediators so they are independent. Can be applied when there is A-$\boldsymbol{M}$ interaction in the outcome model.
SPCMA (8)	Point, interval estimation	No	Identifies whether subsets of the mediators are jointly active	Continuous	No	Similar to PCMA but applies sparse PCA, resulting in transformed mediators that are more interpretable.
HILMA (9)	Point, interval estimation	No	No	Continuous	No	Uses a debiased penalized regression approach to directly estimate the global indirect effect $\boldsymbol{\alpha}_a^T\boldsymbol{\beta}_m$. Can be applied for multiple exposures simultaneously.
PMED (10)	Point, interval estimation	No	Yes	Continuous	Yes	Estimates β_a by fitting the outcome model with the mediator effects penalized by the SCAD penalty. Then estimates $\boldsymbol{\alpha}_a^T\boldsymbol{\beta}_m$ by subtracting the estimated β_a from an estimate of the total effect.
Group 3 Methods						
HDMM (11)	No	No	Nonspecifically identifies groups of active mediators	Continuous	No	Estimates "directions of mediation" by which the observed mediators can be linearly combined to form latent mediators. The latent mediators replace the true mediators in the analysis. Requires $p < n$[1]
LVMA (12)	No	No	Identifies inputted mediators associated with latent mediators	Continuous or binary	Yes	Reformulates the causal structure of the mediation problem. Assumes that $\boldsymbol{M}$ itself is not responsible for mediation, but rather that the effect of A on Y is mediated by latent, unmeasured factors, $\boldsymbol{F}$, which also cause changes in $\boldsymbol{M}$.

[1] The other methods can be used with more mediators than observations. However, the methods that allow covariates require fewer covariates than samples.

References

1. Zhang H, Zheng Y, Zhang Z, Gao T, Joyce B, Yoon G, et al. Estimating and testing high-dimensional mediation effects in epigenetic studies. Bioinformatics. 2016 Oct 15;32(20):3150–4.
2. Gao Y, Yang H, Fang R, Zhang Y, Goode EL, Cui Y. Testing Mediation Effects in High-Dimensional Epigenetic Studies. Front Genet. 2019;10:1195.
3. Zhang Q. High-Dimensional Mediation Analysis with Applications to Causal Gene Identification. Stat Biosci. 2021;14:432–51.
4. Zhao Y, Luo X. Pathway LASSO: pathway estimation and selection with high-dimensional mediators. Stat Interface. 2022;15(1):39–50.
5. Song Y, Zhou X, Zhang M, Zhao W, Liu Y, Kardia SLR, et al. Bayesian shrinkage estimation of high dimensional causal mediation effects in omics studies. Biometrics. 2020 Sep;76(3):700–10.
6. Song Y, Zhou X, Kang J, Aung MT, Zhang M, Zhao W, et al. Bayesian sparse mediation analysis with targeted penalization of natural indirect effects. J R Stat Soc Ser C (Applied Stat. 2021 Nov 1;70(5):1391–412.
7. Huang Y-T, Pan W-C. Hypothesis test of mediation effect in causal mediation model with high-dimensional continuous mediators. Biometrics. 2016 Jun;72(2):402–13.
8. Zhao Y, Lindquist MA, Caffo BS. Sparse principal component based high-dimensional mediation analysis. Comput Stat Data Anal. 2020;142:106835.
9. Zhou RR, Wang L, Zhao SD. Estimation and inference for the indirect effect in high-dimensional linear mediation models. Biometrika. 2020 Sep 1;107(3):573–89.
10. Guo X, Li R, Liu J, Zeng M. High-Dimensional Mediation Analysis for Selecting DNA Methylation Loci Mediating Childhood Trauma and Cortisol Stress Reactivity. J Am Stat Assoc. 2022 Jul 3;117(539):1110–21.
11. Chén OY, Crainiceanu C, Ogburn EL, Caffo BS, Wager TD, Lindquist MA. High-dimensional multivariate mediation with application to neuroimaging data. Biostatistics. 2018;19(2):121–36.
12. Derkach A, Pfeiffer RM, Chen T-H, Sampson JN. High dimensional mediation analysis with latent variables. Biometrics. 2019 Sep 1;75(3):745–56.
