# Supplementary material for: Methods for mediation analysis with high-dimensional DNA methylation data: Possible choices and comparisons
Source: PLoS Genet. 2023 Nov 7;19(11):e1011022. doi: 10.1371/journal.pgen.1011022 (PMC10655967; doi:10.1371/journal.pgen.1011022)
Supplement: S2 Table — (PDF) [file pgen.1011022.s013.pdf]

S2 Table. Complete list of primary simulation settings.

Number of potential mediators (p)	Sample Size (n)	Sparsity of signals	Degree of correlation	PVE_A	PVE_{IE}	PVE_{DE}
2000	2500	Sparse	Baseline	0.20	0.10	0.10
2000	2500	Sparse	Baseline	0.20	0.05	0.10
2000	2500	Sparse	Baseline	0.10	0.10	0.10
2000	2500	Sparse	Baseline	0.20	0.10	0.05
2000	2500	Sparse	High	0.20	0.10	0.10
2000	2500	Sparse	High	0.20	0.05	0.10
2000	2500	Sparse	High	0.10	0.10	0.10
2000	2500	Sparse	High	0.20	0.10	0.05
2000	2500	Non-sparse	Baseline	0.20	0.10	0.10
2000	2500	Non-sparse	Baseline	0.20	0.05	0.10
2000	2500	Non-sparse	Baseline	0.10	0.10	0.10
2000	2500	Non-sparse	Baseline	0.20	0.10	0.05
2000	1000	Sparse	Baseline	0.20	0.10	0.10
2000	1000	Sparse	Baseline	0.20	0.05	0.10
2000	1000	Sparse	Baseline	0.10	0.10	0.10
2000	1000	Sparse	Baseline	0.20	0.10	0.05
2000	1000	Sparse	High	0.20	0.10	0.10
2000	1000	Sparse	High	0.20	0.05	0.10
2000	1000	Sparse	High	0.10	0.10	0.10
2000	1000	Sparse	High	0.20	0.10	0.05
2000	1000	Non-sparse	Baseline	0.20	0.10	0.10
2000	1000	Non-sparse	Baseline	0.20	0.05	0.10
2000	1000	Non-sparse	Baseline	0.10	0.10	0.10
2000	1000	Non-sparse	Baseline	0.20	0.10	0.05

PVE_A: Percent of variance in Y explained by the exposure. PVE_{IE}: Percent of variance in Y explained by the indirect effect. PVE_{DE}: Percent of variance in Y explained by the direct effect.
