# Supplementary material for: Methods for mediation analysis with high-dimensional DNA methylation data: Possible choices and comparisons
Source: PLoS Genet. 2023 Nov 7;19(11):e1011022. doi: 10.1371/journal.pgen.1011022 (PMC10655967; doi:10.1371/journal.pgen.1011022)
Supplement: S3 Table — (PDF) [file pgen.1011022.s014.pdf]

S3 Table. Complete list of additional simulation settings.

Modification	Number of potential mediators (p)	Sample Size (n)	Sparsity of signals	Degree of correlation	PVE_A	PVE_{IE}	PVE_{DE}
Non-negative Effects	2000	2500	Sparse	Baseline	0.20	0.10	0.10
Non-negative Effects	2000	2500	Sparse	Baseline	0.20	0.05	0.10
Non-negative Effects	2000	2500	Sparse	Baseline	0.10	0.10	0.10
Non-negative Effects	2000	2500	Sparse	Baseline	0.20	0.10	0.05
Unmeasured Confounding (Variance of confounder = 1)	2000	2500	Sparse	Baseline	0.20	0.10	0.10
Unmeasured Confounding (Variance of confounder = 2)	2000	2500	Sparse	Baseline	0.20	0.10	0.10
Unmeasured Confounding (Variance of confounder = 3)	2000	2500	Sparse	Baseline	0.20	0.10	0.10

PVE_A: Percent of variance in Y explained by the exposure. PVE_{IE}: Percent of variance in Y explained by the indirect effect. PVE_{DE}: Percent of variance in Y explained by the direct effect.
