# Supplementary material for: Methods for mediation analysis with high-dimensional DNA methylation data: Possible choices and comparisons
Source: PLoS Genet. 2023 Nov 7;19(11):e1011022. doi: 10.1371/journal.pgen.1011022 (PMC10655967; doi:10.1371/journal.pgen.1011022)
Supplement: Attachment — Submitted filename: response_to_reviewers.pdf [file pgen.1011022.s015.pdf]

Response to Reviewer Comments

Title: Methods for mediation analysis with high-dimensional DNA methylation data: Possible choices and comparisons

Authors: Clark-Boucher, Dylan; Zhou, Xiang; Du, Jiacong; Liu, Yongmei; Needham, Belinda L; Smith, Jennifer A; Mukherjee, Bhramar

Ref: PGENETICS-D-23-00581

We would like to thank the editorial board and reviewers for their constructive feedback on our previous submission, and for giving us the opportunity to address their concerns through a revised manuscript. Our itemized responses to the editor and reviewer feedback are listed below. The original comments are in plain black text, our responses are in green, and the changes to the manuscript are in *italicized green*. We hope that you find the revised manuscript suitable for publication in *PLOS Genetics*.

Response to the Editor

As the authors can see the reviewers raised some important points, but appreciate the usefulness of the findings and the addressed topic. Beyond their comments, I'd like to ask the authors to address the point of unmeasured confounders: in the simulations it is always assumed that there is no unmeasured confounder and the linear model fit reflects causation. Also, what happens if variables treated as mediators are actually colliders? Such simulation scenarios (including model misspecifications) and the presence of unmeasured confounding factors should be explored and commented on in the Discussion.

Response: Thank you for your important comments. We have addressed the issue of collider bias by highlighting this point in the Discussion section, and we have addressed the issue of unmeasured confounding by implementing additional simulations with an unmeasured confounder. See below.

1. Collider bias: In a case where *M* is a collider, not a mediator, the causal path involving *M* becomes $A \rightarrow M \leftarrow Y$. Within this framework, alpha*beta no longer represents the mediation effect of that mediator, but rather the collider effect, for a continuous *Y* and *M* [1]. When confronted with collider variables, people tend to prioritize estimating the total effect of *A* on *Y* [2], rather than understanding the decomposition of the pathway effect from *A* to *Y*.

However, if there are multiple variables treated as mediators instead of a single *M*, and some of those variables are in fact colliders, the estimate of the global mediation effect has the potential to be extremely biased, depending on factors such as the ratio of colliders to mediators, the strength of the collider and mediation effects, the correlation structures of the variables, and whether the active and inactive mediators affect the colliders themselves. To our knowledge, this type of setting has not been explored in the high-dimensional mediation analysis literature or been a focus of recent methodological work. Thus, though we agree that collider bias would be an interesting topic for future simulation studies in the high-dimensional mediation analysis context, we do not feel these simulations would fall within the scope of our manuscript, since the objective of our work is to contrast the different methods in commonly-considered scenarios. In response to this issue, we have added the following text to our Discussion section:

Text added to Discussion:
"Second, the validity of the mediation analysis depends on the strong assumption that the causal mechanism is correctly specified—that is, that the exposure affects the mediators, that the mediators affect outcome, and that confounding of this relationship is accounted for by the model. If there is unmeasured confounding of the causal pathway, or if some of the variables treated as mediators are, in fact, colliders, the parameters of the high-dimensional mediation model become difficult to interpret, and the estimate of the global indirect effect may be highly biased. Though recent work by [3] has directly considered the issue of unmeasured confounding in a high-dimensional mediation model, the issue of collider bias is an important area for future research."

2. Confounder bias: To address the issue of unmeasured confounding, we ran additional simulations in which there is a latent confounder affecting the exposure, outcome, and mediators. A directed acyclic graph (DAG) of this mechanism is shown in Figure R1. The simulations begin with our baseline simulation setting, with n = 2,500, PVE_A = 0.2, PVE_{IE} = 0.1, and PVE_{DE} = 0.1, then perturb the data-generating mechanism by adding a confounding variable, *U*, that is ignored when we perform the mediation analysis. To see how the results change as the level of confounding increases, we set the sensitivity analysis parameter, namely the variance of *U* to either be 1, 2, or 3. The effects of the confounder on *A*, $\boldsymbol{M}$, and *Y* are held constant.

An explanation of this procedure has been added to the Methods section. Since one of the reviewers also recommended additional simulations, the new simulation scenarios with unmeasured confounding are described throughout the Methods section under the label "Additional simulation settings". The explanations we have added are included below.

Results for the unmeasured confounding simulations are included in Figure R2. While the true positive rate for detecting active mediators is somewhat robust to unmeasured confounding, the mean-squared error for estimating mediation contributions and the percent relative bias for inferring the global mediation effect increase quickly as the variance of the confounder increases. However, the *relative* mean-squared error for estimating the mediation contributions, relative to the one-at-a-time method, improves as the confounding becomes more severe, which implies that the one-at-a-time mediation method is substantially less robust to confounding than the high-dimensional methods. This observation reaffirms the importance of using methods that are explicitly designed for multiple mediators, rather than assessing the mediators independently. Figure R2 has been added to the manuscript supplement. Commentary on the results has been added to the Discussion; see below.

Text added to Methods:

*"Finally, the second additional scenario considers data-generating mechanisms in which there is an unmeasured confounding variable, U, that directly influences the exposure, the outcome, and a subset of the mediators. For these simulations, we begin with Setting (1) (as described above) with the first set of signal strength parameters (PVE_A = 0.2, PVE_{DE} = 0.1, PVE_{IE} = 0.1), then perturb the data-generating mechanism by adding confounding effects of U to the generation of A, **M**, and Y. We explore different levels of confounding by setting the sensitivity analysis parameter, namely the variance of U, to be 1, 2, or 3, while holding the effects of U on the other variables constant. In both additional simulation scenarios we set* n *to be 2,500. We provide a list of the additional scenarios in the supplement (S3 Table). Results for both scenarios are reported in the supplement as well (S1-S2 Figs)."*

"For the unmeasured confounding simulations, the modified data-generating mechanism is described in models (3), (4), and (5), which are shown below:

$$A_i = \gamma U_i + \delta_i \qquad (3)$$

$$\tilde{M}_i = \tilde{\alpha}_a A_i + \tilde{\alpha}_u U_i + \tilde{\epsilon}_i \qquad (4)$$

$$Y_i = \beta_a A_i + \beta_u U_i + \tilde{\beta}_m^T \tilde{M}_i + \zeta_i \qquad (5)$$

Here, δ_i and ζ_i are independent normal random variables with mean zero, and their variances are chosen to be equal to their values from the baseline setting (1 and s^2, respectively). In model (4), $\tilde{\epsilon}_i$ is a multivariate normal random vector, independent of δ_i and ζ_i, with variance-covariance matrix set to be S from the baseline setting. The confounder-exposure effect γ is set to be 1/3, and the confounder-outcome effect β_u is set to β_a/2. For the vector of confounder-mediator effects, $\tilde{\alpha}_u$, we set the jth entry to be $(\tilde{\alpha}_a)_j/2$ if $(\tilde{\alpha}_a)_j$ is not zero, and set it to be 1/2 if $(\tilde{\alpha}_a)_j$ is zero but $(\tilde{\beta}_m)_j$ is not zero. (That is, only the mediators that are affected by A, affect Y, or both, are

directly affected by U.) The choice of the these fractions (e.g., $\beta_a/2$) is somewhat arbitrary, but it ensures the confounding effects are on a similar scale to the coefficients of interest, only slightly weaker. The remaining parameters are held at their values from Setting (1). The confounding variable U is sampled from a normal distribution with mean zero and variance τ, the sensitivity analysis parameter, which is set to be 1, 2, or 3. This varies the intensity of the confounding.

Text added to Discussion describing simulation results:

In simulation scenarios with an unmeasured confounder, the performance of the multiple-mediator methods became worse as the severity of the confounding effects increased, in terms of estimating the global mediation effect or inferring the mediation contributions. However, the relative performance of these methods compared to the one-at-a-time approach improved substantially with more confounding, which emphasizes the importance of evaluating the mediators simultaneously rather than one-by-one.

[1] MacKinnon, D. P., & Lamp, S. J. (2021). A unification of mediator, confounder, and collider effects. *Prevention Science*, *22*(8), 1185-1193.

[2] Cole, S. R., Platt, R. W., Schisterman, E. F., Chu, H., Westreich, D., Richardson, D., & Poole, C. (2010). Illustrating bias due to conditioning on a collider. *International journal of epidemiology*, *39*(2), 417-420.

[3] Wickramarachchi DS, Lim LHM, Sun B. Mediation analysis with multiple mediators under unmeasured mediator-outcome confounding. *Stat Med* [Internet]. 2023 Feb 20;42(4):422–32. Available from: https://doi.org/10.1002/sim.9624

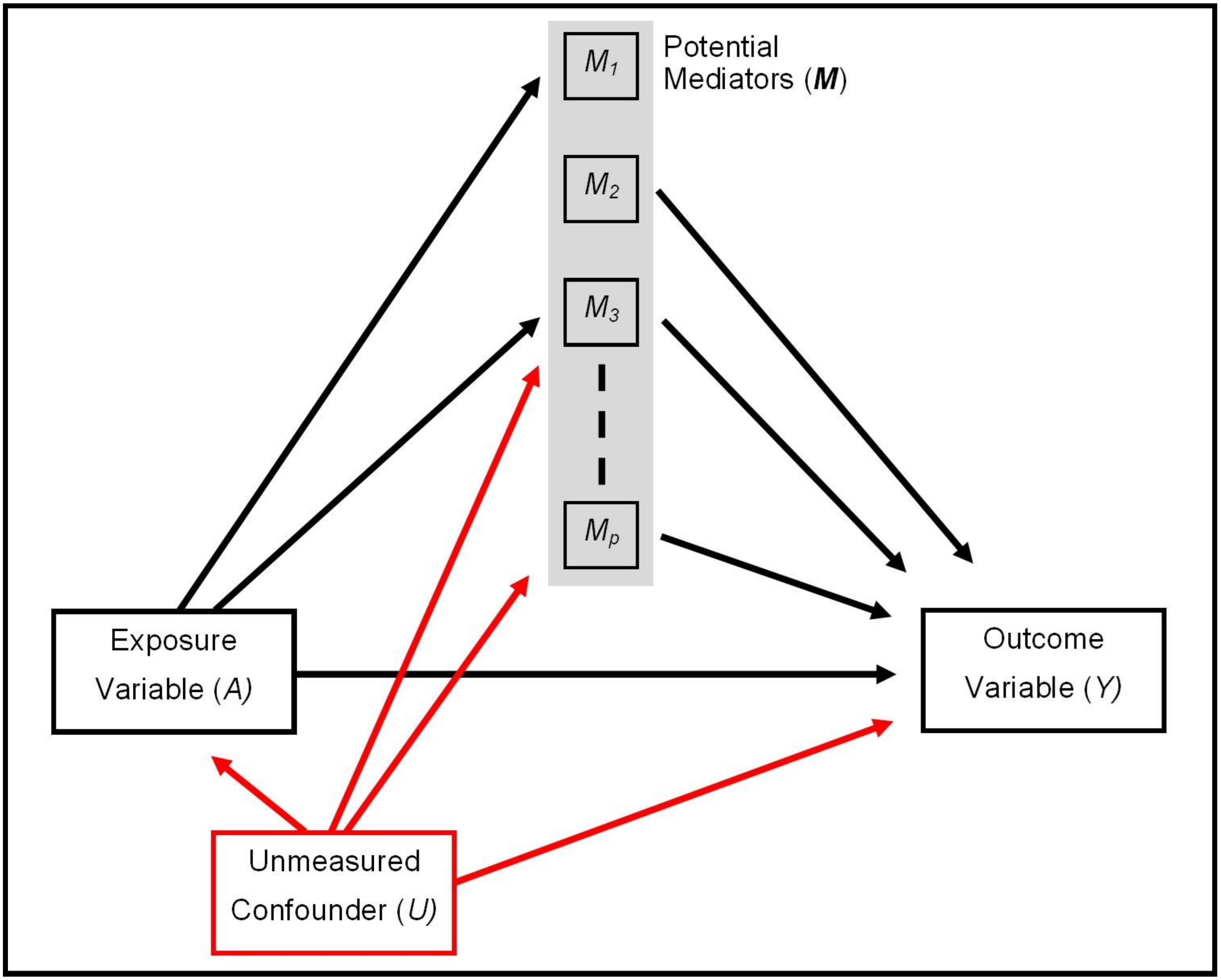

Figure R1: Directed acyclic graph (DAG) showing high-dimensional mediation analysis with an unmeasured confounder. A causal mechanism in which the effect of *A* on *Y* is partially mediated by a subset of the potential mediators, M_1, …, M_p, but there is an unmeasured confounding variable, *U*, that affects *A*, *Y*, and a subset of the potential mediators.

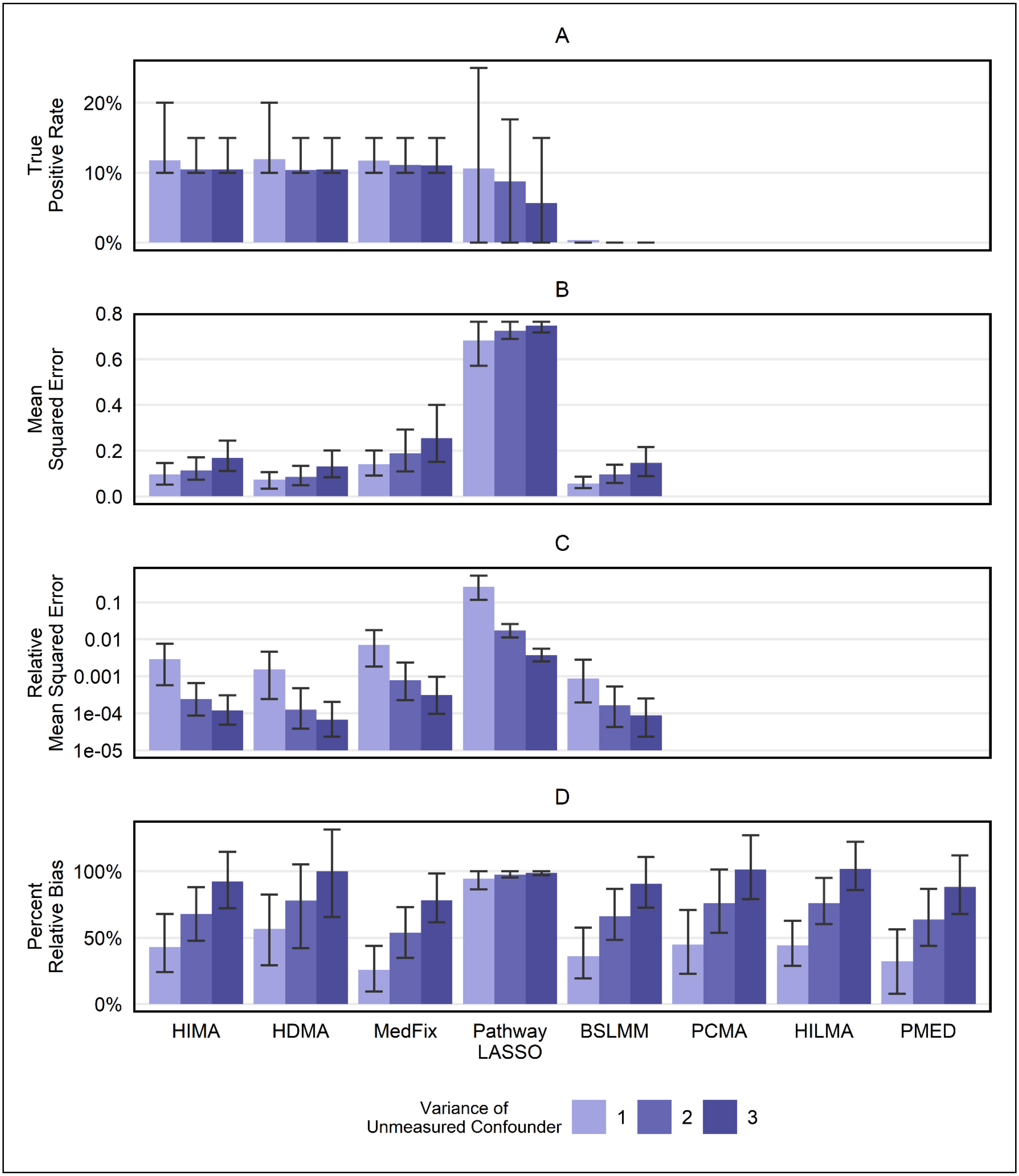

Figure R2: Results for simulations with an unmeasured confounder *U*. (A) True positive rate for detecting active mediators. (B) Mean squared error for inferring the mediation contributions of *active* mediators. (C) Relative mean squared error for inferring the mediation contributions of *active* mediators, relative to the one-at-a-time method. (D) Percent relative bias for inferring the global mediation effect.

Responses to Reviewer 1

The manuscript is a timely evaluation and comparison of the methods for high dimensional mediation with a single exposure, continuous mediators and continuous outcome. The R package is also a valuable resource for the practitioners working with high dimensional mediation analysis. While the paper focuses on methylation data, it is expected to benefit the researchers in other areas as well. The following are my specific comments.

1.Correlation: the issue of "correlations" or correlated mediators is mentioned in many places in this manuscript. The mediators may be correlated for different reasons. They can be causally related, or their noise terms may be correlated, or they are conditionally independent given the exposure and the confounders, and merely marginally correlated due to the exposures and the confounders. The authors did not make any distinction between them, which may unintentionally mislead the readers. One such example (along many) is line 94-96 on page 5. It reads "Instead, so that we leverage these correlations rather than ignore them, the preferred approach is to evaluate the mediators jointly through a single, multivariable statistical model." This is largely not true. Using the multivariate outcome model only addresses the marginal correlation due to their dependence on common exposure and confounders. Most of the surveyed methods assume that the mediators are not causally interdependent, and some of the inference procedures further assume independent noise for mediators. The authors need to clarify what types of correlations that these methods can "leverage", and what their common limitations are.

Response: We thank the reviewer for raising this important point. As suggested, there are many types of correlations that can appear in multivariate data analysis, and we apologize for any lack of clarity in the explanation we provided before. We have updated the text in numerous places to clarify the description of the correlations.

In general, the methods included in our paper consider settings in which the mediators result from the exposure plus random noise. The correlations explored in our simulations are those which occur in the noise term; for example, if mediator M1 and mediator M2 were assigned to be correlated, they would be simulated as $A\alpha_1 + \epsilon_1$ and $A\alpha_2 + \epsilon_2$, respectively, where the epsilon terms are normally distributed with the specified correlation. This is the same framework considered by the methods HIMA, HDMA, pathway LASSO, BSLMM, GMM, PCMA, SPCMA, and HILMA. (The exception is MedFix, for which the original paper appears to treat the mediators as independent conditional on the exposure and covariates.)

This specific type of correlation makes it advantageous to fit a multivariable outcome model, rather than separate mediator-specific outcome models, because the marginal correlations among mediators can only partially be explained by A and C. If the marginal correlations between mediators were due only to the exposure and covariates, one could simply fit a different outcome model for each mediator and arrive at the same linear estimates as produced by the multivariable outcome model. However, when there is also correlation in the random noise term, the mediators will still be correlated after conditioning on A and C, making the results from the single-mediator outcome models potentially biased.

To help clarify this issue throughout the text, we have changed the wording in several places, shown below. (Note that the page numbers correspond to the revised manuscript, not the original manuscript).

Revised text:

Page 5, line 109: *Moreover, although a naïve strategy in such settings would be to evaluate the potential mediators one-at-a-time, in separate models, this approach can be problematic when the mediators are correlated conditional on the exposure variable and covariates, since the resulting estimates may be biased due to confounding from the co-mediators that were excluded. There could also be loss in efficiency due to lack of exploiting the joint multivariable structure. To reduce the risk of bias and to increase precision, it is better to evaluate the mediators jointly and fit a single, multivariable outcome model that adjusts the effect of each mediator for the others, rather than fitting multiple one-at-a-time models.*

Page 16, line 331: *"(1) a baseline setting in which the mediation signals are sparse and the error terms of the (potential) mediators are moderately correlated"*

Page 17, line 370: "*In Settings (1) and (3), we tune S so that the error correlations between mediators range from -0.37 to 0.49, and in Setting (2), so that they range from -0.68 to 0.89."*

Page 21, line 463: "*We consider (1) a baseline setting, where the error terms of the mediators are moderately correlated and the signals of the mediators are sparse; (2) a high-correlation setting, where the error correlations between mediators are enhanced compared to (1); and (3) a non-sparse setting, where every mediator has at least some mediation signal but some of the signals are systematically larger.*"

2.Interpretations of the mediation contribution: This is partially related to the correlation issue. The authors have presented the causal assumptions for the total mediation effect but not the mediation effect of the individual mediators. Instead, they say that they cannot be interpreted as a causal effect through the jth mediator. This is true in general. But it will be more helpful if the authors can present the assumptions under which alpha_j*beta_j can be interpreted as a causal effect through the jth mediator. I believe that some of the surveyed papers have presented such assumptions. The authors also need to clarify further the meaning of "mediation contribution" and its limitations. For example, if a mediator's mediation effect is completed mediated by other mediators, it may not be significant in the outcome model, but it is still "active" somewhere in the causal network among the mediators.

Response: We thank the reviewer for addressing the specific meaning of the mediation contribution, which we agree is an important point to clarify in the text. We have addressed this point by making changes to the referenced paragraph; see below.

Revised text: *Lastly, we may also seek to measure the mediator-specific product terms* $(\tilde{\alpha}_a)_j(\tilde{\beta}_m)_j$*, which we will call the* mediation contributions*. The mediation contribution of the* j^{th} *mediator reflects the mathematical contribution of that mediator to the global mediation effect, since the sum of* $(\tilde{\alpha}_a)_j(\tilde{\beta})_j$ *over all j equals* $\tilde{\alpha}_a^T\tilde{\beta}_m$*. These parameters are intuitive to estimate, but difficult to interpret. Though it is tempting to refer to* $(\tilde{\alpha}_a)_j(\tilde{\beta}_m)_j$ *as a causal effect corresponding to the jth mediator, we emphasize that this parameter cannot generally be interpreted as the natural indirect effect through that mediator specifically. Identifying the indirect effects of specific mediators, in settings with multiple mediators, requires strong assumptions about whether the group of mediators are sequentially ignorable—conditions that would be violated, for example, in situations where a subset of the mediators have causal effects on some of the others. (The exact assumptions are not described here as they would require a discursion into counterfactual inference. See [4]). Despite the limited interpretability of the mediation contributions, we will describe a mediator as* inactive *if its mediation contribution is zero, and* active *otherwise. This has the caveat that if a mediation contribution is zero, that mediator could still be involved in the causal path from A to Y, since complex causal relationships among the set of mediators might exist.*

[4] Imai, K. and Yamamoto, T. (2013). Identification and sensitivity analysis for multiple causal mechanisms: Revisiting evidence from framing experiments. Political Analysis, 21(2):141–171.

3.The simulation setting appears to resemble a setting with inconsistent mediation in which half of the mediators have positive mediation effects and the other half have negative mediation effects, and they partially cancel each other out. While it is common in multivariate mediation analysis, it will be better to include a case where the mediation effects of the individual mediators are more consistent. It means that the direct effect, total effect, and the mediation effects of most true mediators have the same sign.

Response: We thank the reviewer for their helpful suggestions for enhancing our simulation study. To clarify, while it is true that $(\widetilde{\alpha}_a)_j$ and $(\widetilde{\beta}_m)_j$ have mixed signs, and therefore $\widetilde{\alpha}_a^T\widetilde{\beta}_m = \sum_j (\widetilde{\alpha}_a)_j(\widetilde{\beta}_m)_j$ will involve some cancellation, the cancellation is not enough for the global indirect effect to become negligible. In fact, in the baseline and high-correlation settings, the global indirect effect was always equal to approximately 6.70, and in the non-sparse setting, approximately 8.06. (The $(\widetilde{\alpha}_a)_j$ and $(\widetilde{\beta}_m)_j$ parameters were randomly sampled once at the *start* of the simulation study, not *within* each replicate.) Moreover, since the proportion of variance of *Y* explained by the global indirect effect (PVE_{IE}) is fixed, the exact size of the global indirect effect is less important.

However, since we agree that a simulation setting with non-negative model coefficients is interesting, we have performed new simulations which resemble our baseline simulations, but with the $(\widetilde{\alpha}_a)_j$ and $(\widetilde{\beta}_m)_j$ modified to be strictly non-negative by taking their absolute value. An explanation of this setting has been added to the Methods section. Since the editor handling the manuscript also suggested new settings for our simulation study—specifically, settings with unmeasured confounding—both the “non-negative effect” and “unmeasured confounding” analyses have been added to the Methods section, under the label “Additional simulation settings”. Our explanation of the non-negative effect simulations is included below.

Results for these simulations are shown in Figure R3, which has been added to our manuscript’s supplement. The results are consistent with those from the previous simulation settings with mediation effects of different signs (Figures 3-5 in main text, baseline setting). Like before, the strongest method for detecting active mediators is uniformly BSLMM, which also performs well at estimating the mediation contributions. However, as seen previously, the penalized regression methods such as HIMA, HDMA, and MedFix perform better than BSLMM at estimating mediation contributions when the global indirect effect explains only a small proportion of the variance in *Y* (the dark red shade in Figure R3). Finally, the best method for estimating the global indirect effect is once again HILMA, which was also strongest in the previous simulations.

We would also like to acknowledge that there are settings in which the analyst might a’priori know the signs of the model coefficients. In such cases, a reasonable option would be to use sign-constrained optimization [5] when fitting the multivariable outcome model, rather than standard procedures such as the LASSO. We have made a note of this point in our Discussion section.

<u>Text added to Methods</u>

“...we consider cases in which the coefficients of the outcome and mediator models are not mixed in sign, but strictly non-negative (as explained below, the coefficients in the primary simulation settings had both positive and negative signs). The non-negative effect simulations are analogous to simulation Setting (1) above, but with the coefficients of the model converted to their absolute value. They include each of the four signal strength settings explored previously.”

“For the simulations with non-negative effects, the $(\tilde{\alpha}_a)_j$ and $(\tilde{\beta}_m)$ coefficients from Setting (1) are converted to their absolute value. Since this also changes the global indirect effect, $(\tilde{\alpha}_a^T \tilde{\beta}_m)$, we update the direct effect, β_a, to equal $\sqrt{(\tilde{\alpha}_a^T \tilde{\beta}_m)^2 \times PVE_{DE}/PVE_{IE}}$, so that the ratio of the variance of Y explained by the direct effect to the variance of Y explained by the indirect effect is the same as previously. No other parameters used to generate the data are modified; for example, the residual variance of the outcome model (s^2) is the same as before.”

<u>Text added to Discussion</u>

“In simulation settings where the effects are strictly non-negative, BSLMM tended to perform best for detecting active mediators and estimating their mediation contributions, while HILMA was again the strongest method for estimating the global mediation effect.”

“If one has prior knowledge that the signs of the outcome model coefficients are in the same direction, a reasonable approach might be to use sign-constrained optimization rather than standard penalties such as the LASSO [5].”

[5] Meinshausen N. Sign-constrained least squares estimation for high-dimensional regression. Electron J Stat. 2013;7:1607–31.

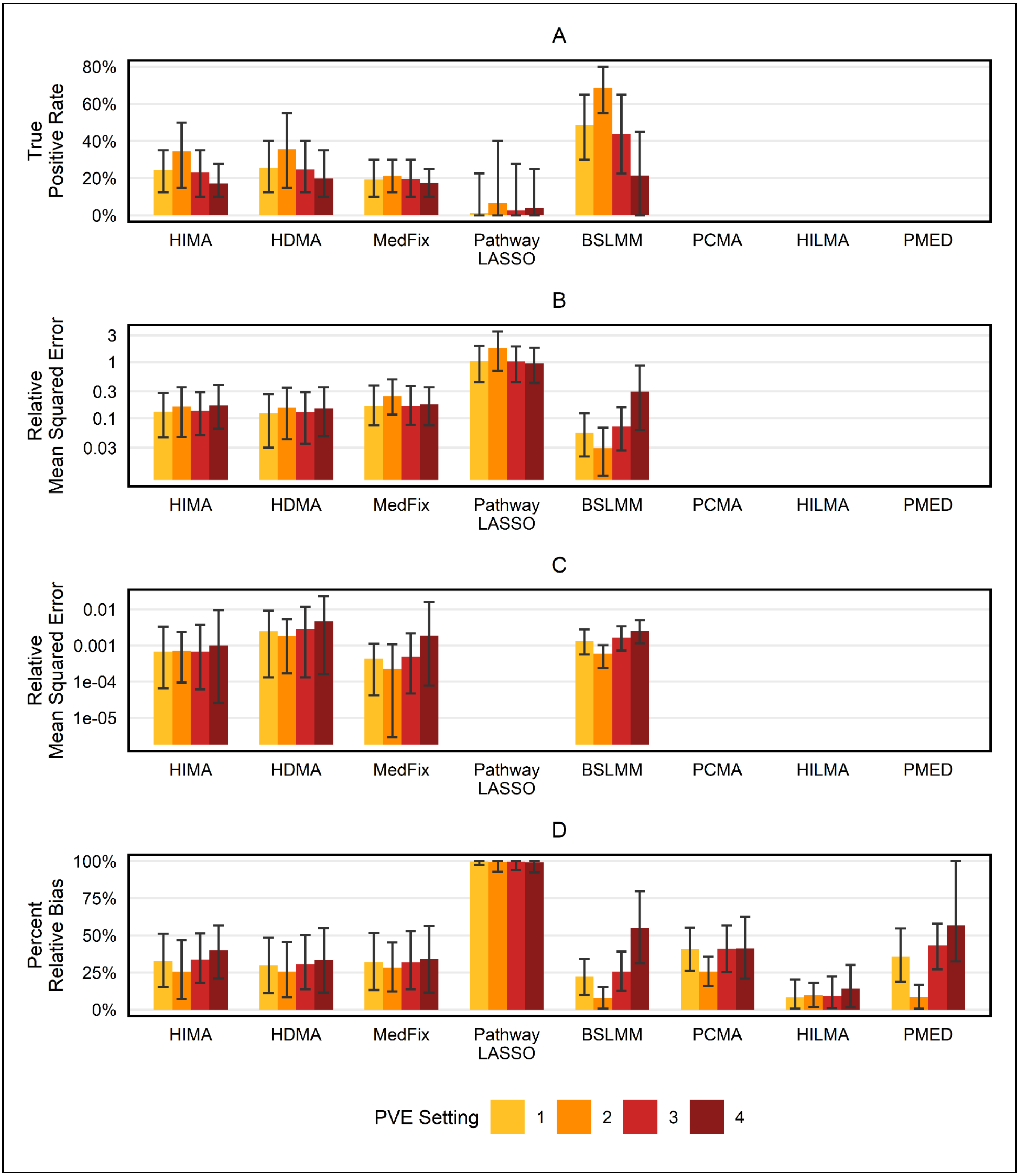

Figure R3: Results for simulations with strictly non-negative effects. (A) True positive rate for detecting active mediators. (B) Relative mean squared error for estimating the mediation contributions of active mediators, relative to the one-at-a-time method. (C) Relative mean squared error for estimating the mediation contributions of inactive mediators, relative to the one-at-a-time method. (D) Percent relative bias for inferring the global mediation effect. The simulation settings were created by taking the absolute values of the

exposure-mediator and mediator-outcome effects in the original baseline simulation settings, which had four different proportion-of-variance-explained (PVE) settings: (1) PVE_A = 0.2, PVE_{DE} = 0.1, PVE_{IE} = 0.1; (2) PVE_A = 0.1, PVE_{DE} = 0.1, PVE_{IE} = 0.1; (2) PVE_A = 0.2, PVE_{DE} = 0.05, PVE_{IE} = 0.1. (4) PVE_A = 0.2, PVE_{DE} = 0.1, PVE_{IE} = 0.05.

4.It will benefit the readers the most, if these methods are evaluated using a real dataset that they have direct access to, instead of behind dbGaP wall.

Response: Thank you for raising the point of data accessibility, which we agree is important for this type of manuscript. We have responded to this point by making changes to our data availability statement that describe how to access the data.

We understand, and lament, that using a non-open access data source is unideal for researchers who may be interested in replicating our findings. However, in light of the substantial DNAm research that has already been published with data from MESA—including many studies with research questions that are similar to ours—we believe that MESA is the strongest choice for the real data analysis portion of this paper. (Note also that due to privacy concerns, it is uncommon for large-scale genetic or epigenetic datasets to be publicly available). In addition, since code for performing the DNAm data analysis with pseudo-data is provided in our GitHub repository (https://github.com/dclarkboucher/mediation_DNAm), and since the methods can be implemented with our R package (https://cran.r-project.org/package=hdmed), our paper's readers should have no issue adapting our work to their own research projects, regardless of whether they choose to retrieve the exact data that was used by our study. For readers who interested in analyzing MESA's data for their own research, a description of how to begin this process has been added to our data availability statement:

Revised data availability statement:

Instructions for generating our simulated data can be found on our GitHub site (https://github.com/dclarkboucher/mediation_DNAm), which includes R-scripts and a ReadMe file that explains how to implement our simulation study. The GitHub site also includes code for implementing our DNAm data analysis, only with pseudo-data instead of the MESA data. The exact data used in the DNAm analysis can be obtained through the MESA Data Coordinating Center (https://www.mesa-nhlbi.org/) (accession: phs000209.v13.p3). Access to MESA's data requires a specific application process depending on the type of project; see https://www.mesa-nhlbi.org/ancillary.aspx and https://www.mesa-nhlbi.org/Publications.aspx for more details.

5.What are the average or median magnitudes of the correlations in the moderate and high correlation simulation settings? They should have meaningful differences.

Response: We again thank this reviewer for their careful attention to our simulation study. We have addressed this comment by modifying the high-correlation setting to make the correlations larger compared to the baseline setting.

As described in the text, the correlations of the error terms of the mediators generated for our simulation study were directly translated from the variance-covariance matrix of the MESA methylation data. Since the correlations we observed were centered close to zero, measures of center such as the mean or median are not helpful metrics for comparing the intensity of the correlations. Instead, we present the percentiles of the *absolute* correlations used in our study in Table R1.

In the table, r is a tuning parameter used to make the variance-covariance invertible, which is beneficial because the data had more mediators than observations, making its covariance matrix singular. Different values of r were used to alter the overall strength of the correlations: In our baseline setting, r was set to 1 so that the residual correlations between mediators were relatively limited. In our original high correlation setting, r was decreased to 0.3 to yield a denser residual correlation matrix with more extremes. However, since we agreed with your concern about the degree of differences between simulation settings, we updated our simulations with r reduced to 0.1, making the variance-covariance very close to that observed on the real data.

All our figures have been updated with the new results, which show slightly greater differences than observed previously. To give an example of this change, see Figure R4 and Figure R5 below, which depict the true positive rate for detecting active mediators *before* and *after* the change to the high-correlation setting, respectively. Examining these figures, it is clear that the high-correlation results are slightly worse across the board with $r = 0.1$ compared to $r = 0.3$, and that the gap between the baseline performance and the high-correlation performance has widened. Since setting $r = 0.1$ leads to similar correlations to what was observed in the real data (Table R1), and since the difference between the high-correlation setting and the baseline setting is prominent (Figure R5), we do not think it is necessary to make the gap in correlations larger. It should be clear to our audience that higher residual correlations among the mediators result in worse performance of the methods.

Table R1. Comparison of Error Correlations between Simulation Settings

Tuning Parameter *r*	Relevance to Paper	Percentiles of the Absolute Correlations (Number of Correlations Greater)			
		50th Percentile (999,500)	75th Percentile (499,750)	90th Percentile (199,990)	95th Percentile (99,950)
0	Real data variance-covariance	0.06	0.11	0.18	0.25
0.1	**Revised** high correlation simulation setting	0.05	0.1	0.17	0.23
0.3	**Original** high correlation simulation setting	0.04	0.08	0.14	0.19
1	Baseline simulation setting	0.03	0.05	0.09	0.12

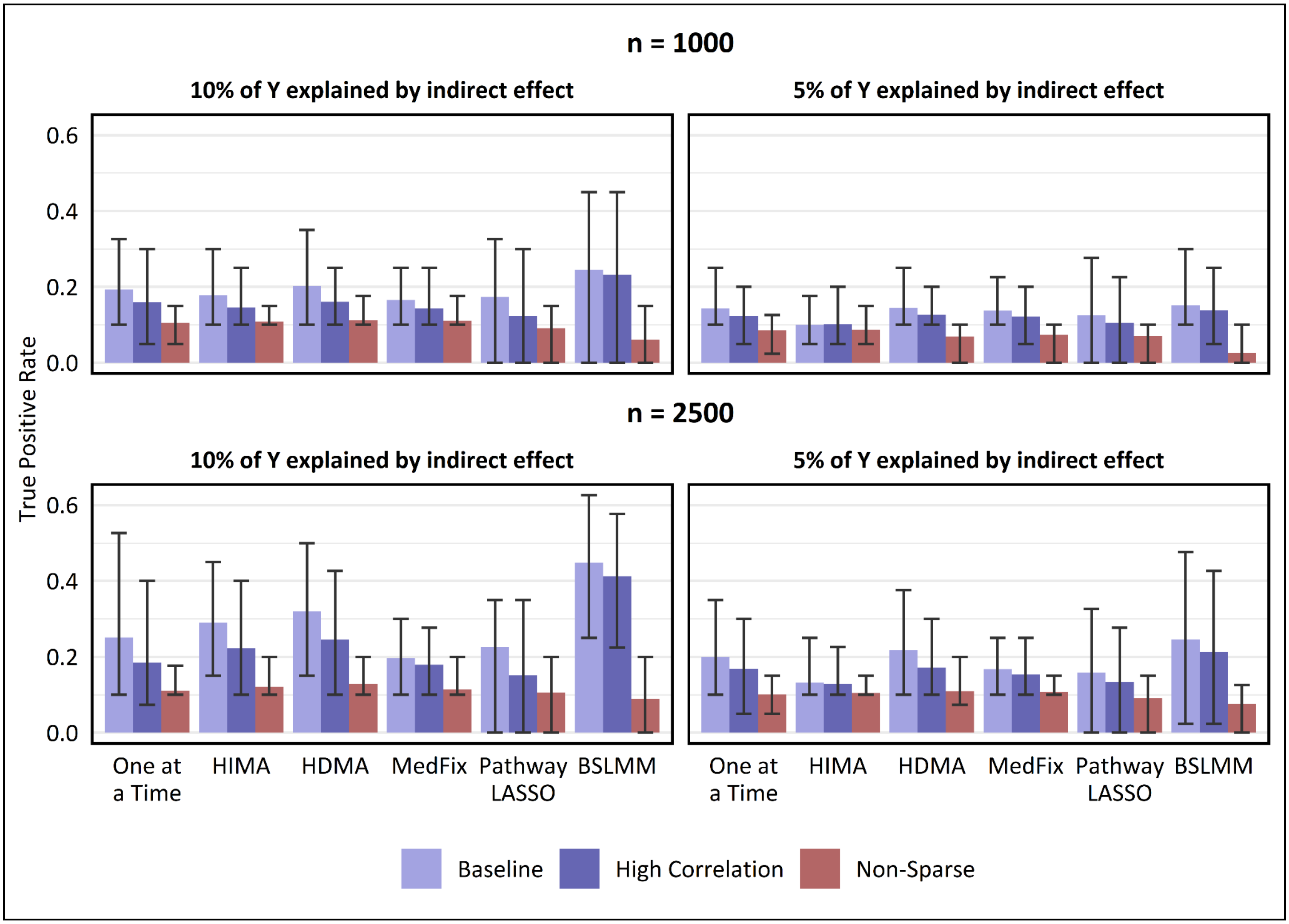

Figure R4: True positive rate for detecting active mediators with the **original** high-correlation setting ($r = 0.3$).

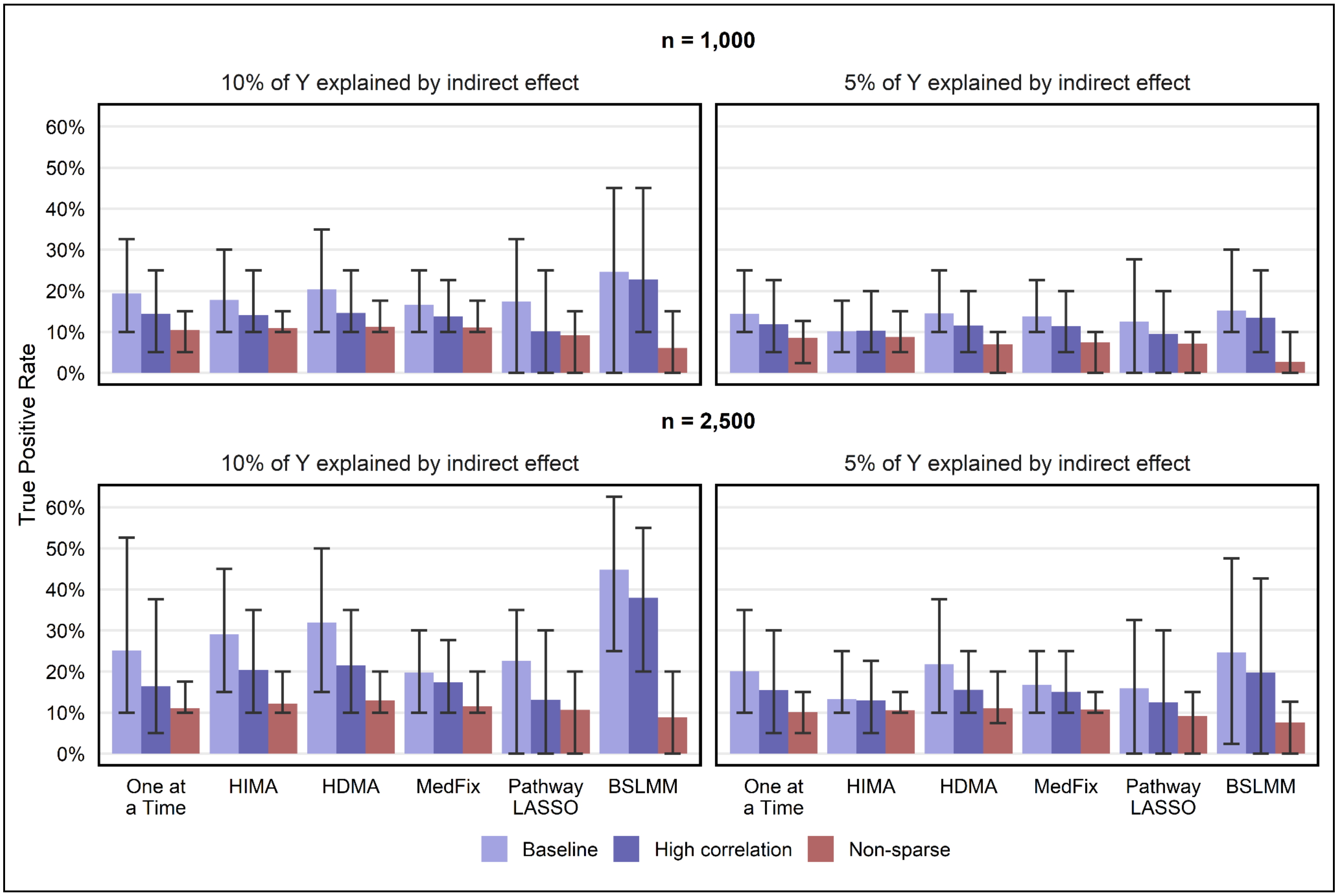

Figure R5: True positive rate for detecting active mediators with the **revised** high-correlation setting (*r* = 0.1)

6.The following paper is a method similar to HILMA published in JASA in 2022. It is faster than HILMA, and the application involves methylation data. It also reports a set of “important mediators” based on the variable selection of the outcome model. But it is not exactly mediator selection and there is no inference for it. They provided code and script to reproduce their results. It is NOT my paper.

Guo, X., Li, R., Liu, J., & Zeng, M. (2022). High-dimensional mediation analysis for selecting DNA methylation Loci mediating childhood trauma and cortisol stress reactivity. Journal of the American Statistical Association, 117(539), 1110-1121.

Response: We thank the reviewer for suggesting this method, which we agree is important to consider in our study. As suggested, we have added this method to our real data analysis and simulation comparison.

To summarize the method briefly, this approach fits a partially-penalized outcome model using the SCAD penalty to obtain an estimated direct effect that is adjusted for the effects of the mediators, which are penalized. The method then estimates the total effect by fitting the outcome model, unpenalized, with the mediators omitted. Finally, the method estimates the global mediation effect by subtracting the estimated direct effect from the total effect. The method also reports “selected” mediators that were chosen by the SCAD-penalized outcome model.

The method has now been added to our paper under the name “partial penalized high-dimensional mediation analysis”, or PMED. Though the method was not given a name in the original manuscript, we feel this name captures a key component of the statistical method while contrasting it from the other methods in our paper. Since the method does not report estimates of the mediation contributions, or provide a method of inference for those contributions, we evaluate PMED solely by its percent relative bias in estimating the global mediation effect, its primary objective. It has also been added to our real data analysis, Figure 2 of the main text, the methods section list of papers, the decision tree in Figure 6 of the main text, the scalability comparison, and supplementary S1 Table. For brevity, we provide only the simulation results and the real data results in this document. You will see that PMED performs competitively in most simulations, with a percent relative bias similar to HIMA, HDMA, and MedFix, but worse than HILMA. However, the performance of PMED suffers when the signal strength is weakened *and* the sample size is small (second row of Figure R6).

On the DNAm data from MESA, fitting the outcome model with the SCAD penalty appears to be more conservative than with the other penalties considered, as none of the potential mediators are selected as having an effect on *Y*. As a result, the global mediation effect estimated by PMED is zero. Though disappointing, this result is not all that different from our results from HIMA, which selected only two mediators and had an estimated global mediation effect of just 0.03. It was a common theme that the methods involving feature selection in the outcome model had smaller estimates of this parameter, most likely because they can set the mediation contributions to be exactly zero.

Added text:

Partial penalized high-dimensional mediation analysis (PMED) is a two-step estimation and inference procedure for the global mediation effect, proposed by Guo et al. (2022). In the first step, the outcome model is fitted with the mediators penalized by the smoothly-clipped absolute deviation (SCAD) penalty. In the second step, the estimated direct effect from the outcome model is subtracted from an estimated total effect, which is obtained by fitting an unpenalized outcome model with the mediators omitted. The method reports the global mediation effect and a set of potentially active mediators selected in step 1, but does not provide estimates of

the mediator-specific mediation contributions. PMED can also be applied when there are multiple, but fewer than n*, exposure variables.*

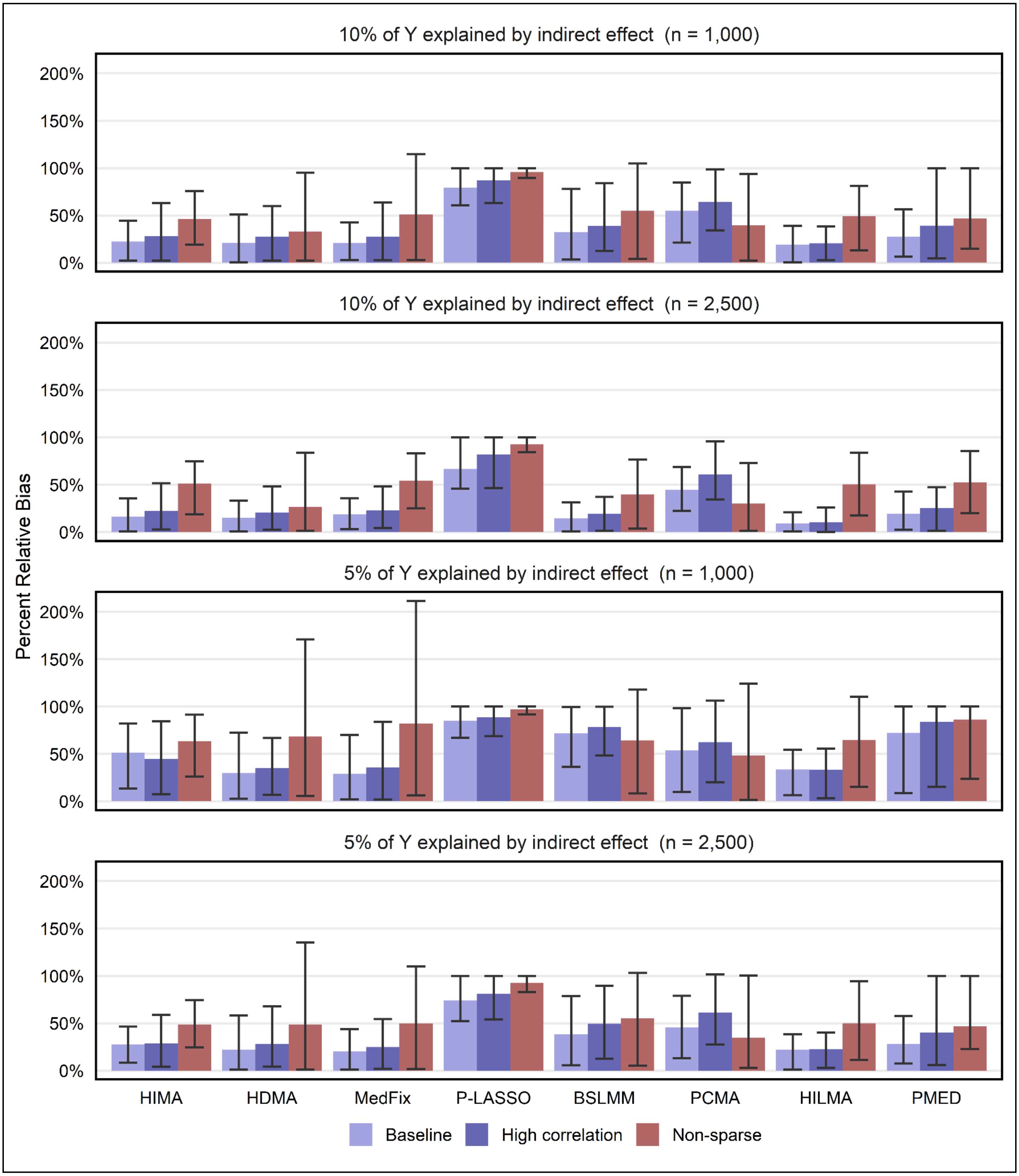

Figure R6: Percent relative bias for estimating the global indirect effect

7.The website for the real data https://www.mesanhlbi.org/ may contain typos. The data availability statement should mention that dbGaP application is needed.

Response: We again thank the reviewer for their interest in how to access MESA's data. You are correct that there was a typo, and we apologize for this error. The correct link is: https://www.mesa-nhlbi.org/. An updated data availability statement is provided above in our response to Question 4.

8.Line 130 on page 6, can p,q be larger than n?

Response: Thank you for helping us clarify the flexibility of the high-dimensional mediation model. Yes, *p* can be larger than *n*, with the exception of the method HDMM, which requires a complex dimension-reduction step to accommodate cases with more mediators than samples. We have revised the text in two places: In line 145 on page 7 (previously, line 130 on page 6), the text has been modified to say *"When p is greater than 1 (and possibly greater than n)"*. In line 317 on page 15, a sentence has been added that explains, "*A limitation of HDMM is that it cannot directly be applied when* p *exceeds* n."

Responses to Reviewer 2

General comment: In this paper, the authors reviewed and evaluated seven statistical methods of mediation analysis using simulations and high-dimensional DNA methylation (DNAm) data. The authors also centralize the computer codes of all the methods into a single, stand-alone R package. In addition, the authors provide some guidelines for the usage of these statistical methods. In particular, the authors created a decision tree for selecting a high-dimensional mediation analysis.

The following are some detailed comments.

Comments:

1. In introduction, the authors said that "Though several methods for fitting such a model have been presented in the literature, none of them are widely used in analyzing DNAm data." However, there are some high-dimensional mediation methods that have been used in DNAm data, e.g., HIMA2 in [1].

Response: We thank the reviewer for helping us clarify: While there are several statistical methods papers that applied their proposed methods to DNAm data—including the papers for HIMA, HIMA2, HDMA, and BSLMM—there has, in the opinion of the authors, been a dearth of studies by practitioners and epidemiologists that have adopted these methods to address their own substantive research questions. The objective of this paper is to encourage and facilitate that adoption in the context of research on DNAm. The text has been revised as follows:

Revised text: *Though several methods for fitting such a model have been presented in the literature, they have yet to be widely adopted by practitioners and researchers for investigating substantive questions on high-dimensional mediation analysis with DNAm.*

2. Moreover, HIMA2 is an extension of the HIMA method. The authors have evaluated the HIMA methods. I was wondering whether HIMA2 will perform better if we add it into comparison.

Response: We thank the reviewer for bringing HIMA2 to our attention, and we agree that this method is important to mention in our manuscript. We have addressed this point by adding a note about HIMA2 in the Methods section of the paper, under the description of HIMA.

Upon examining HIMA2 in detail, there are two main differences between HIMA2 and HIMA. First, the HIMA2 authors use the de-biased LASSO procedure instead of the minimax concave penalty, which improves the inferential step while reducing bias. Second, the authors implement a novel correction procedure to adjust the p-values once the model has been fit, rather than using Bonferroni correction as in HIMA.

The first of these changes makes HIMA2 very similar to HDMA, which was a direct extension of HIMA (by different authors) that replaced the minimax concave penalty with de-biased LASSO. This is why the HIMA2 paper compares HIMA, HDMA, and HIMA2 in its simulation study: HIMA2 only differs from HDMA in its p-value adjustment.

The similarity between HIMA2 and HDMA makes it undesirable to add HIMA2 to our comparison. To make this clearer, recall that in our simulation study, the true positive rate is determined by empirically correcting the p-values to obtain a false-discovery rate of at most 10 percent—a procedure that depends on the raw p-values, not corrected p-values. In addition, our real data analysis does not report p-values or test them against any threshold; we report CpG sites that were *noteworthy* (meaning they had a non-zero mediation contribution), not *significant*. Thus, since p-value adjustment would not change the results of either of our analyses, we feel it is not necessary to include both HIMA2 and HDMA in our analysis.

However, since HIMA2 is a novel method that builds on HIMA, we do feel it is important to mention it in our manuscript. We have added the following text to the paragraph about HIMA:

"*A new version of HIMA, called HIMA2, was published recently. HIMA2 is similar to HDMA, but suggests a p-value correction procedure that maintains the false discovery rate for detecting active mediators. HIMA2 is omitted from our comparison due to its similarity to HDMA.*"

3. In data application, the authors mentioned that they used model (6) to select 2000 CpG sites. But I did not find the model (6) in the paper. Can the authors write or locate the model (6) more clearly?

Response: We thank the reviewer for identifying this error. The reference to a "phantom" model (6) should instead have been a reference to model (4), which is the mediator model with high-dimensional methylation sites as mediators. However, since the paper now defines 3 additional models in the Methods section, the correct reference for this sentence is now model (7), and the text has been updated accordingly.

4. In simulation analysis on Page 16, the authors mentioned not only the MSE in mediation contributions of active mediators but also MSE in mediation contributions of mediators. However, in simulation results, only the MSE in contributions of active
mediators is presented. I was wondering why the MSE in mediation contributions of all mediators is ignored.

Response: We thank the reviewer for noticing this error. Indeed, the phrase which currently reads "the mean squared error in estimating the mediation contributions of mediators" was supposed to say "the mean squared error in estimating the mediation contributions of *inactive* mediators". This line has been corrected in the main text, along with the corresponding formula. As stated in the Results section, the results for inactive mediators are provided in the supplement.

5. In the DNAm data analysis, the authors calculated the estimated mediation contributions of each method. What is a clear definition of the estimated mediation contributions?

Response: We thank the reviewer for raising this point, which is similar to a concern raised by Reviewer 1. See the revised text explaining the mediation contributions below:

<u>Revised text:</u> *Lastly, we may also seek to measure the mediator-specific product terms* $(\tilde{\alpha}_a)_j(\tilde{\beta}_m)_j$*, which we will call the* mediation contributions*. The mediation contribution of the jth mediator reflects the mathematical contribution of that mediator to the global mediation effect, since the sum of* $(\tilde{\alpha}_a)_j(\tilde{\beta})_j$ *over all j equals* $\tilde{\alpha}_a^T\tilde{\beta}_m$. *These parameters are intuitive to estimate, but difficult to interpret. Though it is tempting to refer to* $(\tilde{\alpha}_a)_j(\tilde{\beta}_m)_j$ *as a causal effect corresponding to the jth mediator, we emphasize that this parameter cannot generally be interpreted as the natural indirect effect through that mediator specifically. Identifying the indirect effects of specific mediators, in settings with multiple mediators, requires strong assumptions about whether the group of mediators are sequentially ignorable—conditions that would be violated, for example, in situations where a subset of the mediators have causal effects on some of the others. (The exact assumptions are not described here as they would require a discursion into counterfactual inference. See [4]). Despite the limited interpretability of the mediation contributions, we will describe a mediator as* inactive *if its mediation contribution is zero, and* active *otherwise. This has the caveat that if a mediation contribution is zero, that mediator could still be involved in the causal path from A to Y, since complex causal relationships among the set of mediators might exist.*

[4] Imai, K. and Yamamoto, T. (2013). Identification and sensitivity analysis for multiple causal mechanisms: Revisiting evidence from framing experiments. Political Analysis, 21(2):141–171.

6. In simulations, the authors generated the exposure, mediators, and outcome from continuous distribution. However, the decision tree for selecting a high-dimensional mediation analysis in Figure 6 says that we should choose HDMA and HIMA methods when the outcome is binary. Should we provide some simulations for the performance of the two methods when the outcome is binary?

References
[1] Perera, C., Zhang, H., Zheng, Y., Hou, L., Qu, A., Zheng, C., Xie, K., and Liu, L. (2022). Hima2: high-dimensional mediation analysis and its application in epigenome-wide dna methylation data. BMC bioinformatics, 23(1):1–14.

Response: We thank the reviewer for this comment. We have addressed this point by adding a comment to the Discussion section.

To clarify, we included a binary case in Figure 6 because HIMA and HDMA were the only considered methods that can directly accommodate a binary outcome. (Note that in supplementary table S1, there is a column for acceptable *Y* data types). Simulations comparing HIMA and HDMA for the case of binary *Y* have already been performed by Gao et al. (2019) in the original HDMA manuscript [6]. Although HDMA generally has stronger performance than HIMA, both methods are listed in the figure because they are both defensible choices.

In general, there are few choices for methods for high-dimensional mediation analysis with generalized linear models rather than least squares. This is an important direction for future research. We have added the following sentence to the Discussion section: "*In terms of data type, methods that can accommodate non-continuous Y and M are in general scarce, and represent an important direction for future methodological development.*"

[6] Gao Y, Yang H, Fang R, Zhang Y, Goode EL, Cui Y. Testing Mediation Effects in High-Dimensional Epigenetic Studies. Front Genet. 2019;10:1195.
